# Supplementary material for: Alginate microencapsulation of an attenuated O-antigen mutant of Francisella tularensis LVS as a model for a vaccine delivery vehicle
Source: PLoS One. 2022 Mar 11;17(3):e0259807. doi: 10.1371/journal.pone.0259807 (PMC8916679; doi:10.1371/journal.pone.0259807)
Supplement: Attachment — Submitted filename: Response to reviewers (Autosaved).docx [file pone.0259807.s003.docx]

Response to reviewers

Reviewer 1

Pg. 4: revised as suggested

Pg 15: Corrected.

P values that are not applicable have been removed from the figure legends.

Reviewer 2

The number of times key experiments were done have been added as well as clarification of the number of animals used.

**Major points**

Fig. 5A. The reason survival is >100% at 1 hour incubation is because the bacteria grew in highly bactericidal serum, indicating they were well protected by the beads. Each bar represents the mean ± the standard error of the mean (SEM). Clarification of how SEMs were calculated and the number of experiments done has been added. Three independent experiments/replicates were run in duplicate per time point (3 separate times with duplicates (mechanical replicates) for each). There are no SEMs at 0 hours because 100% of the bacteria were viable in all experiments and groups t that time. Asterisks and brackets have been added where relevant. Three independent experiments were run in duplicate per experiment. This has been added to the Materials and Methods and to the legend. The figure is more quantitative than the table. However, the APA beads could not readily be dissolved like the alginate-only beads, and therefore is qualitative only. Therefore, we could only incubate the beands in complement-active serum for the indicated period and assay for viable bacteria. Fig. 5A shows the quantitative results for alginate beads and 5B also shows the qualitative results for bacteria within APA beads. Fig. 5B shows that the bacteria could survive longer in the APA beads than in alginate alone (24 and 48 hrs). Therefore, we would prefer to keep the revised Fig. 5A and 5B as is. Encapsulated in Fig. 5A refers to alginate alone, and has been clarified in the legend.

Fig. 6 and legend. I presume the reviewer is referring to LVS here as the “mock immunized”, but the “mock immunized” mice received only buffer and made no antibody response. The number of mice used in these vaccine trials have now been added to the Methods section and the legend. Four mice (2 male and 2 female) were used in each vaccine group is order to keep the numbers of mice used to the minimum number that can yield statistically significant results, as required by IACUC. The number of mice that were not immunized (mock) were 2, also to keep the number of control mice used to a minimum. Four mice were immunized with WbtI APA beads, IP. There is no SD on this column because all the mice made antibody titers greater than 1:12,800 and that was as far as the sera was tittered out. This information has been added to the legend. The mock-immunized mice were inoculated IP. Some mock-immunized mice were inoculated SQ, but these data were not shown because there was no difference from those inoculated IP. I cannot say that the titers of the mock-immunized mice were 0, but they were below the level of lowest dilution (1:100). This information has been added to the legend. The antibody titers of mice inoculated with WbtI and WbtI APA SQ were not determined. LVS was not inoculated IP because there is no sublethal dose for that strain via that route. However, the emphasis of this figure is on the comparison of how the addition of LPS or LVS in APA beads affects the immune response. The data are representative of 3 separate experiments carried out in duplicate, so each value is the mean of 6 experiments. The values represent the mean titer ± the standard error of the mean. The APA vaccine groups are not statistically different from the non-APA vaccine group (at least WbtI+LPS vs WbtI+LPS APA are not significantly different titer-wise). The only significant difference is between the Mock vs WbtI+LPS and Mock vs WbtI+LPS APA when the analysis was done using the more rigorous non-parametric analyses due to the relatively small number of individuals/ group (4). However, when reanalyzed using ANOVA with tukey’s test post-hoc all formulations were significantly greater than mock-immunized, except WbtI + LPS SQ, and all formulations inoculated IP were significantly greater than the same formulations inoculated SQ. As mentioned in the text, the immune response to *F. tularensis* within alginate only beads was not measured because WbtI_G191V_ survived significantly longer in beads with the APA coating. As we were seeking the optimal immune response and use of the minimal number of animals, non-APA vaccines were not examined.

Fig. 7. As stated above, each vaccine group consisted of 4 mice (2 male and 2 female). The experiment was not repeated because the results were not that dramatically different. The data are representative of the experiment done.

Fig. 8. The histopathology consisted of 4 mice per IP and SQ groups, resulting in 42 samples analyzed, and read blindly. This information has been added to the Methods section and the legend.

Page 25, 13-2; Fig. 9 and legend: There was a typo in this sentence and “subcutaneously” has been changed to intraperitoneally” in the results, and inoculation with LVS clarified to be SQ. The reference to “9a” has been corrected to “9b”. “9b” has been corrected to “9c”. Fig. 9 has been redone and additional data have been added to it. Three-to four samples were analyzed and read of each tissue from each of the 4 mice, resulting in 42 total samples being read and analyzed. This information has been added to the legend. The values are represented on a scale of 0-3, 0 = no inflammatory infiltrates present. Grading of 1-3 represent increasing amounts of cellular infiltrates and are based on two references that have been added to the manuscript. This information has been added to the Methods section.

Fig. 10 legend: As described in the Methods section, RNA was isolated from each sample of each animal, and then pooled by immunization group. This information has been added to the legend.

**Minor points**

Pg. 4: Revised as suggested.

Page 15, line 4: There is no reference needed at this specific site. The reference to Fig. 1 has been moved to where it is relevant.

Page 17, lines 3-4; Fig 2.: Fig. 2 has been revised to include asterisks and brackets to show difference in significance.

Page 17, line 6, 16 and page 18, line 17: spacing around “Fig. 3” has been corrected.

Fig. 3: titles of x and y axis have been revised as suggested.

Page 11, line 16: oral immunization was planned originally , but due to technical problems was not done. Mention of oral immunization has been taken out of the methods section.

Page 24, lines 6-14: Although the data are reported in the text, the statement “data not shown” has been added, as the data are not shown in a table or figure.

Fig. 6 legend and M&M: We have added that the antibodies detected are total IgG (H&L chains).

Figs. 8 and 9: “spleen, liver and lung” have been added to the appropriate panels and the terms for the material used for immunization has been made consistent with that in Fig. 6.

Page 28, line 13: That LVS is no longer licensed has been changed to LVS is not licensed.
